# *Helicobacter pylori* healthy South Asians

**DOI:** 10.1002/jgh3.12426

**Published:** 2020-10-12

**Authors:** Sanjeev Kharel, Anil Bist, Suraj Shrestha, Sushan Homagain

**Affiliations:** ^1^ Maharajgunj Medical Campus Tribhuvan University Institute of Medicine Kathmandu Nepal

**Keywords:** asymptomatic infection, epidemiology, *Helicobacter pylori* infection, South Asia

## Abstract

We aimed to estimate the pooled prevalence of *Helicobacter pylori* among asymptomatic South Asians based on available literature and highlight the importance of screening asymptomatic individuals and implementing preventive strategies for eradicating *H. pylori*. Electronic databases such as PubMed and Embase, a regional database of WHO South Asian Region, and gray literature sites were searched for relevant studies from 1983 to 5 May 2020. In addition, references of the included studies were thoroughly searched. The random‐effect model was used to calculate the pooled prevalence with a 95% confidence interval (CI) along with subgroup analysis. Analysis of 19 studies showed a pooled prevalence of 56.5%, ranging from 10.3 to 91.7%. In subgroup analysis by country, the highest prevalence rate was reported from Bangladesh (86.3%, 95% CI: 0.806–0.921), whereas the lowest prevalence was from Sri Lanka (10.3%, 95% CI: 0.072–0.135). No differences were found between males and females. Prevalence among children and adolescents was 65.3% (95% CI: 0.529–0.777), greater than adults, 56.9% (95% CI: 0.353–0.785). The prevalence rate showed a decreasing trend upon comparison of studies conducted before and after 2000. Our analysis reveals the high prevalence of *H. pylori* infection among asymptomatic healthy populations in South Asia, particularly in children and adolescents. Public health awareness and sanitation interventions, pure drinking water, and respective strategies on a policy level to eradicate *H. pylori* and additional extensive multicentric cohort studies are necessary.

## Graphical abstract

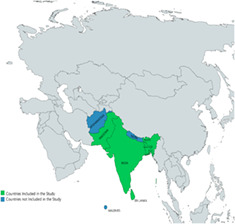

Our article reports the high prevalence of *Helicobacter pylori* infection among the asymptomatic populations of South Asia, especially in children and adolescents compared to adults, but shows a decreasing trend. Public health interventions and timely screening are necessary to eradicate *H. pylori* and its complication.

## Introduction

*Helicobacter pylori* is a gram‐negative, nonspore‐forming spiral bacteria colonizing the human stomach with a global prevalence.^1^ Approximately 4.4 billion individuals in 2015 were estimated to be positive for *H. pylori* infection with/without any apparent signs or symptoms.^2^ Of these, virtually all develop some form of gastritis, but only a small proportion develops clinical manifestations of infection, including gastric and duodenal ulceration, gastric noncardia adenocarcinoma, and gastric mucosa‐associated lymphoid tissue lymphoma.^3^ Thus, *H. pylori* is considered a class I carcinogen and the most important risk factor for developing gastric cancer, the timely elimination of which could lead to a 75% reduction in risk.^4^

High rates of *H. pylori* prevalence have been reported from developing countries (85–95%) compared to that of developed countries (30–50%).^5^, ^6^, ^7^ In contrast to the western countries, Asian countries have a high prevalence of *H. pylori* infection and severe gastroduodenal disease along with gastric neoplasm.^8^ Moreover, developing countries like India, Pakistan, Bangladesh, and Thailand have a higher frequency of *H. pylori* infection compared to industrialized and developed regions like Japan, China, and South Korea. In addition, there is a marked difference of *H. pylori* infection between and within populations of different Asian countries.^8^, ^9^

Most *H. pylori* infections are acquired in early childhood (30–50%) but can reach over 90% during adulthood in developing countries, largely attributed to poor socioeconomic status and overcrowded conditions.^2^, ^10^, ^11^, ^12^

Countries of South Asia include a group of developing countries. They are classified either as lower‐middle‐income or low‐income countries. South Asian countries share similarities in race, economy, health‐care status, education, and culture, which differ from other Asian countries. Lack of proper sanitation and delivery of safe drinking water, improper hygienic conditions, overcrowding, the highest rate of open defecation worldwide, and low socioeconomic conditions, all of which are considered to be risk factors for *H. pylori* infections, are prevalent in South Asia.^2^, ^10^, ^11^, ^12^ South Asian countries have a high prevalence of *H. pylori* infection, considered a major public health burden.^2^, ^13^

A recent meta‐analysis reported a prevalence rate of 44.7% (39.4–50) in Asia and 57.7% (49.9–65.5) in South Asia.^14^ The South Asian region in particular has had very few summaries of *H. pylori* infection prevalence, but no systematic review and meta‐analysis have been conducted to date. A need for a rigorous review to estimate the hidden burden of *H. pylori* infection is necessary for this region. As most of the infected individuals are asymptomatic carriers, this will eventually manifest as chronic gastritis under gastroscopy.^15^, ^16^ Besides, due to the relationship between *H. pylori* and gastric cancer, it is important to pay attention to the asymptomatic population. Thus, we aimed to conduct a systematic review and meta‐analysis to evaluate the prevalence of *H. pylori* among healthy populations in South Asia and to identify its differences across countries, age groups, gender, and time course.

## Methods

### *Study protocol*

The study protocol, with well‐defined methodology and inclusion criteria, was registered on PROSPERO with reference number ID: CRD42020183544, with a published link available: https://www.crd.york.ac.uk/prospero/display_record.pH.pylori?ID=CRD42020183544.

#### *Literature search and Study selection*

We conducted an electronic literature search of the PubMed and Embase databases and other supplementary sources of articles published in the English language with human participants within the time frame of 1 June 1983 to 22 May 2020. In addition, a manual search was conducted of journals along with WHO South Asian databases like Index Medicus for Southeast Asia Region and other repositories of gray literature. The references of included studies were also thoroughly searched for any remaining studies. Authors of some studies were contacted via email and ResearchGate for retrieval of full texts and clarification of doubts. Articles were screened from 1 June 1983 to 1 May 2020. The predefined search terms were identified to form a comprehensive search strategy that included all fields within records and Medical Subject Headings (Mesh terms) for expanding the search in an advanced PubMed search. Mesh of related terms; Boolean operators (AND, OR); and “*Helicobacter pylori*,” “*H. pylori*,” “epidemiology,” “prevalence,” “seroprevalence,” “incidence,” and “frequency” keywords were used for the systematic identification of records. The names of South Asian countries—Afghanistan, Bangladesh, Bhutan, India, Maldives, Nepal, Pakistan, and Sri Lanka—were combined with search terms. The preliminary search strategy is given in Appendix 1, Supporting information.

Studies obtained from the electronic databases, supplementary sources, manual searching, gray literature sites, and other repositories were exported to ENDNOTE reference software version 9.1 (Thomson Reuters, Stamford, CT, USA) in compatible formats. Duplicate articles were screened first by ENDNOTE and then manually. Duplicates were then recorded and removed. The titles and abstracts of the studies remaining after duplicates were removed were screened independently by four authors (Sanjeev Kharel, Anil Bist, Suraj Shrestha, and Sushan Homagain). Two authors (Sanjeev Kharel and Anil Bist) retrieved the full text of potentially eligible studies and further screened for final inclusion. Disagreements between reviewers were resolved by a third author (Suraj Shrestha), and the final consensus was reached. For multiple publications of the same data in more than one journal, the most inclusive, comprehensive studies, with a larger sample size, and the most recent ones were considered.

**Figure 1:**
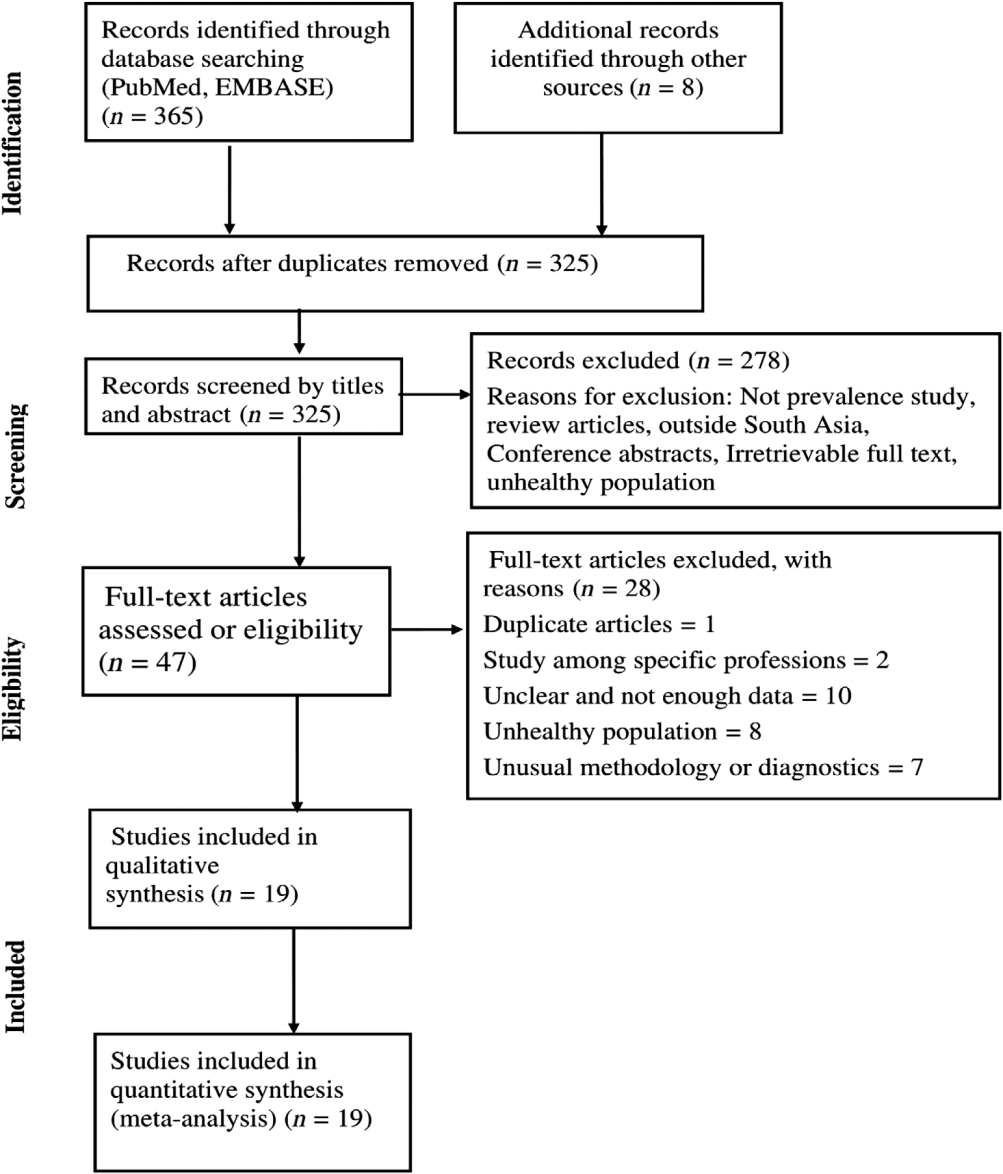
Preferred Reporting Items for Systematic Reviews and Meta‐Analyses diagram detailing the study identification and selection process.

The systematic review and meta‐analysis were guided by the Preferred Reporting Items for Systematic Reviews and Meta‐Analyses (PRISMA) guidelines. The PRISMA diagram detailing the selection process is shown in Figure 1.

### *Eligibility criteria*

#### *Inclusion criteria*

Healthy/asymptomatic population (regardless of age, gender, and ethnicity)‐based (cross sectional, cohort, or case–control) studies published since 1983 reporting *H. pylori* infection in South Asian countries were considered for inclusion. Only studies in which *H. pylori* infections were confirmed by one of the following tests: *H. pylori* serology, *H. pylori* stool antigen, urea breath test, and biopsies for Campylobacter‐like organism test, rapid urease test, histology, or culture, were included in the review.

#### *Exclusion criteria*

Editorials, case reports, systematic review, meta‐analysis and conference abstracts, studies including participants with a high prevalence of *H. pylori* infection (patients with gastric cancer, peptic ulcer, and other gastrointestinal disorders, patient with cancer and human immunodeficiency virus) and high‐risk population groups (refugees, prisoners, homeless people, adoptees), articles with irretrievable full texts (after requesting full texts from the corresponding authors via email and ResearchGate for 2 weeks), records with unrelated outcome measures, and articles with missing or insufficient outcomes were excluded. Besides, research that did not report our outcome of interest was excluded after reviewing (by all four authors).

#### *Data extraction*

Full‐text review of the selected articles was performed, and data were retrieved using the standard data extraction format in Microsoft Excel version 2013 (Microsoft Corp., Redmond, WA, USA) under the variables: author name, year, country, location (city, region), event, sample, numbers by gender, age distribution, method of diagnosis, and prevalence (or frequency) of *H. pylori* infection in available studies. The prevalence rate, if not mentioned, was calculated relative to the sample size wherever appropriate.

#### *Quality assessment*

To evaluate the quality of the studies included in this review, the Newcastle‐Ottawa Scale for cross‐sectional studies and case–control studies was used (Appendix 2). Age and gender as confounding factors with probability sampling were considered for all observational studies. The follow‐up period was considered to be up to 6 months for cohort studies. Using the tool as a checklist, the qualities of each of the original articles were evaluated independently by the authors (Sanjeev Kharel and Anil Bist). Interrater reliability (Cohen's kappa) was calculated to assess the level of agreement between two authors in the quality assessment of the studies. The mean score of two authors was taken for the final decision, and articles (≥5 of 10 across the three parts) were included in the analysis. The detailed quality assessment of the articles is shown in Appendix 3.

#### *Statistical analysis*

The data collected in the Excel sheet were exported, and analysis was performed using the STATA software version 16 (StataCorp, College Station, TX, USA). Prevalence estimates of *H. pylori* infection were calculated by pooling the study‐specific estimates with its 95% confidence interval using the random‐effects model by Der Simonian and Laird's random‐effects model.^17^ Heterogeneity of *H. pylori* infection prevalence of the included studies was examined using the Cochrane Q test and I^2^ statistic (I^2^ of less than 25% defined as mild heterogeneity, I^2^ of 25–50% as moderate heterogeneity, and I^2^ of more than 50% as severe heterogeneity).^18^ Sensitivity analyses were performed by serially excluding each study to determine the effect of individual studies on the degree of heterogeneity (Appendix 4). Subgroup analyses for *H. pylori* infection prevalence by country, gender, age group (<18 years old as children and adolescent and ≥18 years old), and study years were carried out when data were available. In order to assess the publication bias, Egger's‐regression tests were performed for the small study effects' sizes and presented with funnel plots of effect size and standard error.^19^ A *P*‐value of <0.10 was considered indicative of a statistically significant publication bias.

## Results

### *Study selection and characteristics*

A total of 373 articles, with 365 obtained from the search of electronic databases and 8 from other additional sources, were identified, of which 48 articles were removed after duplication. After reviewing 325 articles, 278 articles were removed after screening titles and abstracts as they did not meet inclusion criteria, and full‐text articles were irretrievable. The remaining 47 full‐text articles were assessed thoroughly, of which 28 articles were excluded because of predefined inclusion criteria and vague or insufficient results and unclear methodology. Finally, 19 studies were included for qualitative analysis (systematic review) and then quantitative analysis (meta‐analysis) after completing the quality assessment.

**Figure 2:**
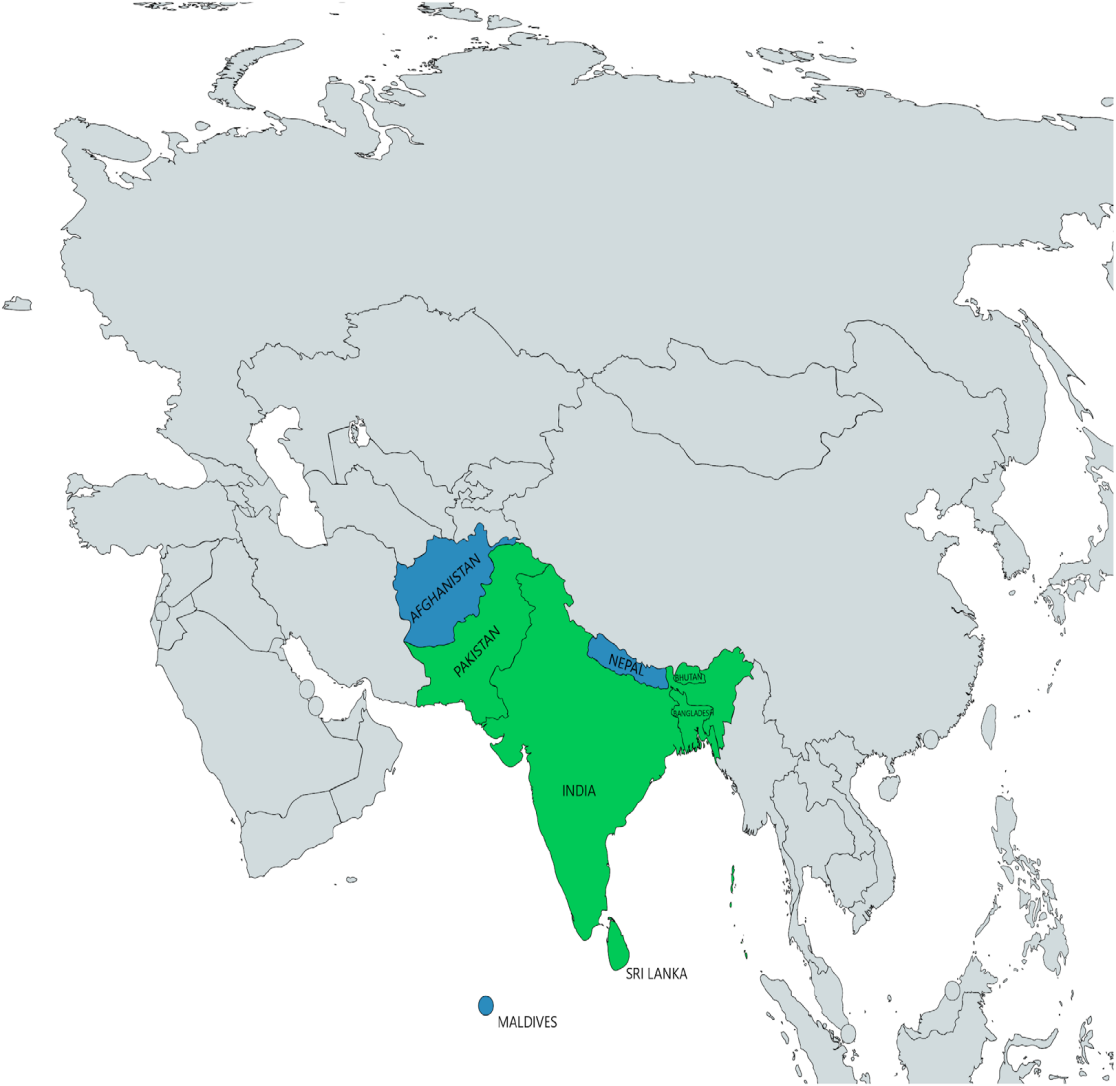
Countries of the South Asian region and those included in the study. (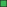), Countries included in the study; (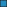), countries not included in the study.

A total of 9614 participants included in 19 studies were investigated for *H. pylori* infection. In the included studies shown in Table 1, the sample size ranged from 30 to 3846 and publication year from 1994 to 2019. Of 19 studies, 12 studies were cross sectional, and the remaining were case–control studies. Nine studies used *H. pylori* serology, five studies used the urea breath test, and only one study used either stool antigen test or histology as diagnostic methods. In two studies, more than one diagnostic method was used. Seven studies reported prevalence among adults, and nine studies reported on children and adolescents. These studies were performed in South Asian countries (Afghanistan, Bangladesh, Bhutan, India, Maldives, Nepal, Pakistan, and Sri Lanka) and are illustrated in Figure 2. No studies from three countries (Afghanistan, Nepal, and the Maldives) of South Asia qualified for the review, while several articles were retrieved from other countries like India (*n* = 8), Pakistan (*n* = 6), Bangladesh (*n* = 3), Bhutan (*n* = 1), and Sri Lanka (*n* = 1). Detailed characteristics of the included studies are summarized in Table 1.

**Table 1:** Characteristics of the included studies

Author	Year	Type of study	Location	Country	Event	Sample	Number by gender	Age distribution	Method of diagnosis	Prevalence (%)
Ahmad	1997	CS	Dhaka	Bangladesh	166	181	M‐181 F‐0	20–44 years	Hp serology	166/181 (91.7%)
Ahmad	2008	CS	Islamabad	Pakistan	289	400	M‐208 F‐192	3–16 years	Urea breath test	289/400 (72.3%)
Augustine	2019	CS	Kerala	India	29	99	M‐NA F‐NA	18–60 Years	Hp serology	29/99 (29.3%)
Dore	1997	CS	Karnataka	India	41	50	M‐29 F‐21	6–18 years	Urea breath test	41/50 (82%)
Fernando	2003	CS	Western province	Sri Lanka	37	359	M‐180 F‐179	1–94 years	Hp serology	37/359 (10.30%)
Jafri	2009	CS	Karachi	Pakistan	926	1976	M‐1008 F‐968	1–15 years	Hp serology	926/1976 (46.9%)
Jafri	2013	CS	Karachi	Pakistan	255	540	M‐256 F‐284	1–15 years	Hp serology	255/540 (47.2%)
Mahskar	2010	CC	Pune	India	57	125	M‐62 F‐63	18–83 years	Stool antigen test	57/125 (45.6%)
Mehmood	2014	CC	Islamabad	Pakistan	63	118	M‐63 F‐55	<25–>50 years	Hp serology	63/118 (53.4%)
Prasad	1994	CC	Vellore	India	25	30	M‐NA F‐NA	21–51 years	Histology	25/30 (83.3%)
Priyadarshani	2018	CS	Puducherry	India	29	99	M‐59 F‐40	18–60 years	Hp serology	29/99 (29.3%)
Rasheed	2011	CS	Barakho, Islamabad	Pakistan	384	516	M‐268 F‐248	2–>19 years	Urea breath test	384/516 (74.4%)
Rifat‐uz‐Zaman	2006	CS	Bahawalpur	Pakistan	1756	3846	M‐2344 F‐1502	5–65 years	Hp serology, urea breath test	1756/3846 (45.7%)
Romshoo	1997	CC	Kashmir	India	10	30	M‐25 F‐5	18–65 years	Urease test, histology	10/30 (33.3%)
Sarker	1997	CS	Nandipur, Dhaka	Bangladesh	406	469	M‐NA F‐NA	1–99 months	Urea breath test	406/469 (86.6%)
Sarker	2004	CS	Nandipur, Dhaka	Bangladesh	145	182	M‐90 F‐92	1–5 years	Urea breath test	145/182 (79.7%)
Singh	2002	CS	Chandigarh	India	38	67	M‐NA F‐NA	15–75 years	Hp serology, rapid urease test, histology	38/67 (56.7%)
Tewari	2012	CC	Northern sector	India	76	200	M‐NA F‐NA	30–>85 years	Hp serology	76/200 (38%)
Wangda	2017	CS	Thimphu	Bhutan	216	327	M‐145 F‐182	4–19 years	Hp serology	216/327 (66.1%)

Data are expressed in proportion (%) if possible.

CC, Case–control; CS, cross‐sectional; F, female; M, male, NA, not available.

### *Study quality*

A risk‐of‐bias assessment of all the included studies was carried out using the Newcastle Ottawa Scale, shown in Appendix 2. The value of Cohen's Kappa was found to be 0.859259, which can be considered to be “strong agreement.” Studies with mean scores greater than or equal to seven were considered “low risk,” while studies with a mean score of less than seven were considered “high risk.” Of 19 studies, 10 were “low risk,” and the remaining 9 were “high‐risk studies.” All the studies with a mean score greater than or equal to five were included in the final analysis.

### *The pooled prevalence of H. pylori*

**Figure 3:**
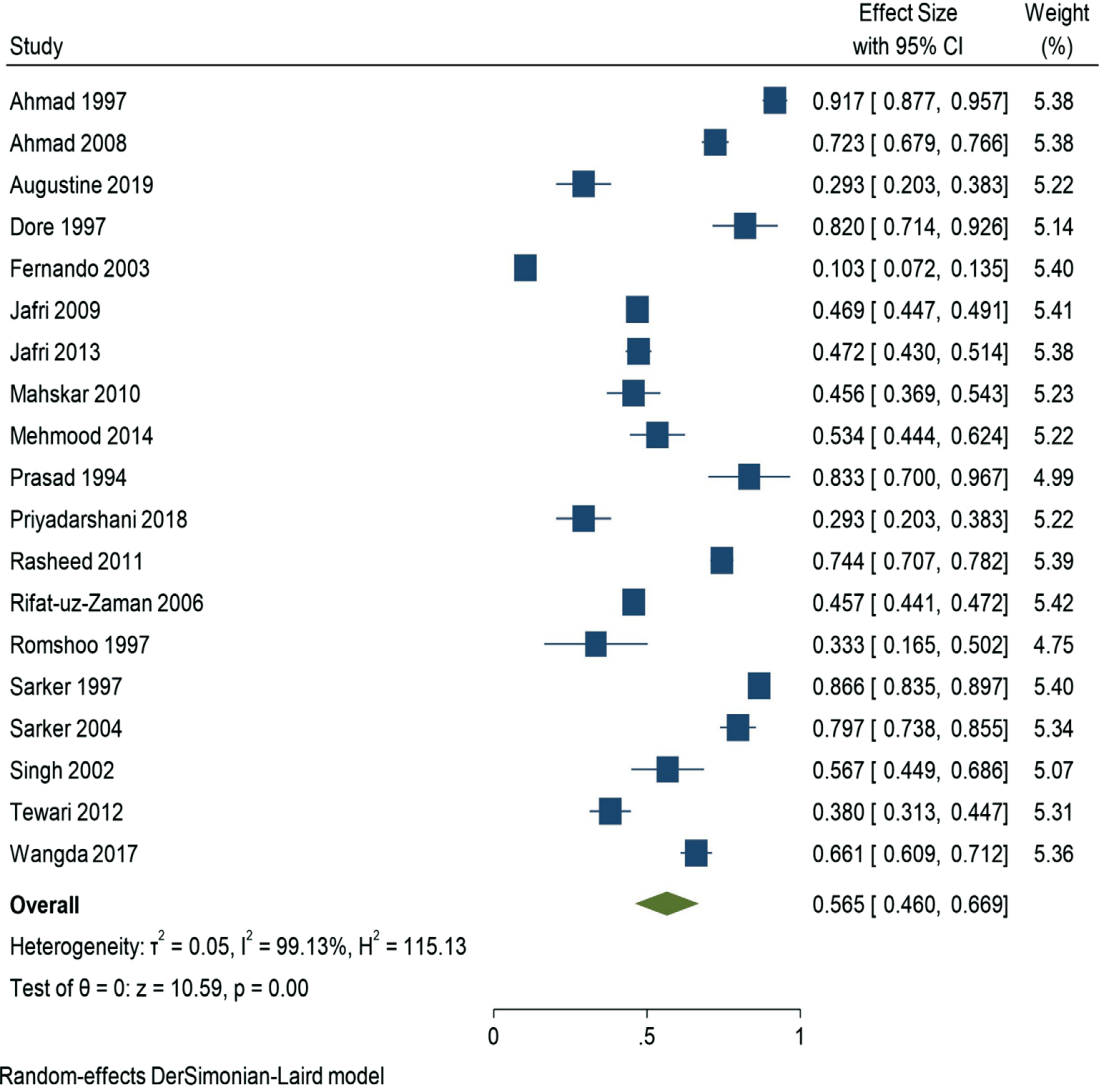
Forest plot showing the estimated pooled prevalence of *H. pylori* observed from 19 studies.

The estimated pooled prevalence of *H. pylori* among the healthy/asymptomatic populations of South Asia observed from 19 studies was 56.5% (95% confidence interval [CI]: 0.460–0.669). We found significant or severe heterogeneity (I^2^ = 99.13%, T^2^ = 0.05, *P* < 0.001) in our study, with prevalence rates ranging from 10.3 to 91.7% (Fig. 3).

### *Sensitivity analysis and subgroup analysis*

**Figure 4:**
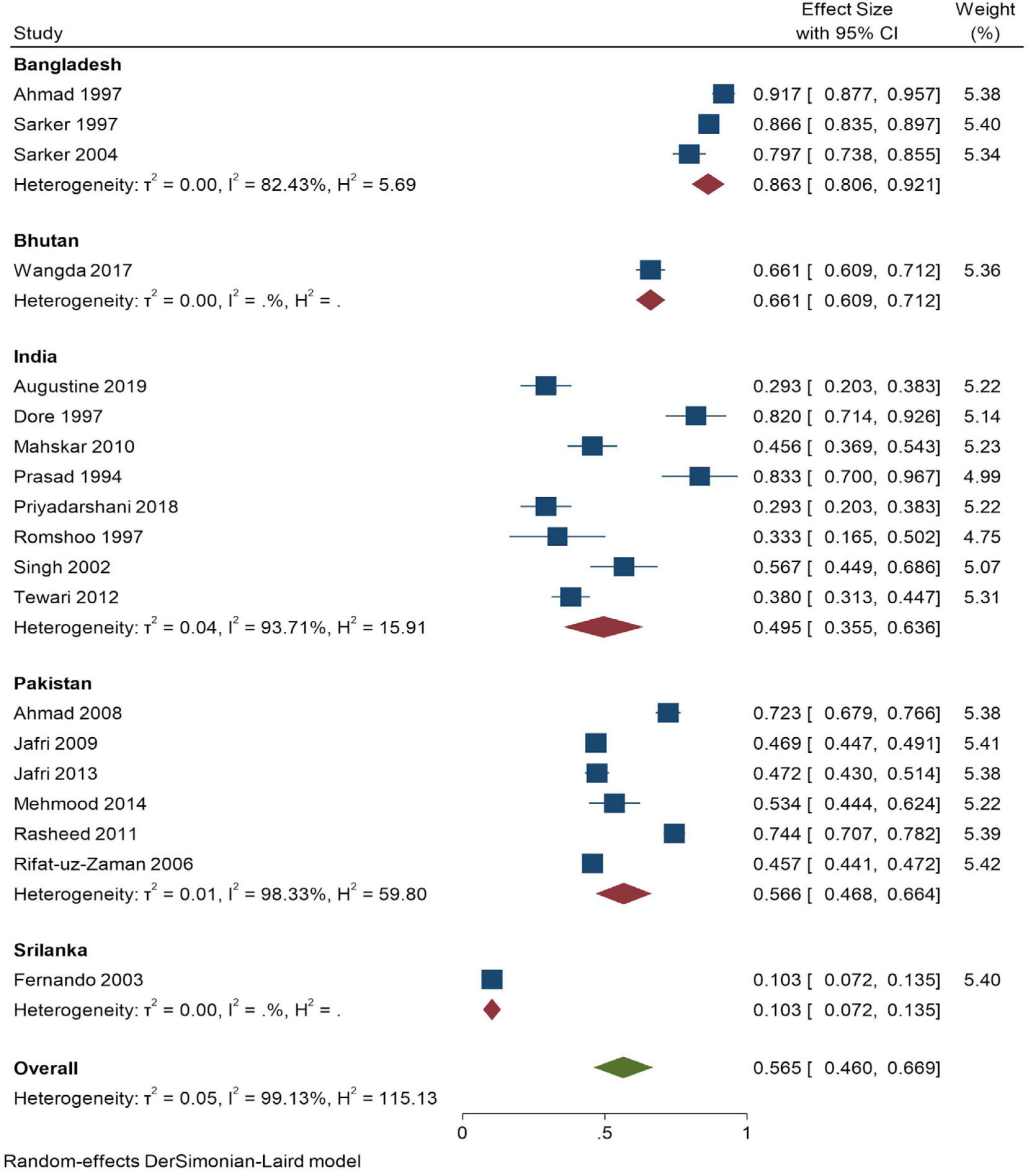
Subgroup analysis of studies showing the prevalence of *H. pylori* infection across countries of South Asia.

There was no significant change in the degree of heterogeneity when studies were omitted one by one in the analysis (Appendix 3). The degree of heterogeneity was between 98.69 and 99.18%. All these studies were included for meta‐analysis. Subgroup analyses for *H. pylori* infection prevalence by country, gender, age group (<18 years old as children and adolescents and ≥18 years old to <60 years old as an adult), and study years were carried out to find the prevalence of *H. pylori*. When analyzed by country, the highest prevalence was found in Bangladesh (86.3%, 95% CI: 0.806–0.921), followed by Bhutan (66.1%, 95% CI: 0.609–0.712) and then Pakistan (56.6%, 95% CI: 0.468–0.664) and India (49.5%, 95% CI: 0.355–0.636), while the lowest prevalence was reported in Sri Lanka (10.3%, 95% CI: 0.072–0.135) as shown in Figure 4. Another subgroup analysis according to gender showed similar prevalence among males (51.3%, 95% CI: 0.393–0.633) and females (51%, 95% CI: 0.406–0.615). Analysis of nine studies showed prevalence among children and adolescents, 65.3% (95% CI: 0.529–0.777), while analysis of seven studies showed the prevalence of 56.9% (95% CI: 0.353–0.785) among adults. Five studies before 2000 showed a prevalence of 78.6% (95% CI: 0.686–0.886), while the remaining studies from 2000 to 2020 showed a prevalence of 49.7% (95% CI: 0.399–0.595) (Table 2).

**Table 2:** *Helicobacter pylori* infection prevalence according to country, gender, age groups, and study years

Study or subgroup	Number of studies	Prevalence (95% CI) (%)	Sample	Heterogeneity test	*P*‐value	Publication bias test
				I^2^ (%)		*P*‐value (Eggers test)
Total	19	56.5 (0.460–0.669)	9614	99.13	<0.001	0.7470
Gender (male)	11	51.3 (0.393–0.633)	4622	98.42	<0.001	0.4944
Gender (female)	11	51 (0.406–0.615)	3734	97.39	<0.001	0.6898
Age group (children and adolescents)	9	65.3 (0.529–0.777)	4285	98.58	<0.001	0.4038
Age group (adults)	7	56.9 (0.353–0.785)	939	98.52	<0.001	0.5312
Study year (before 2000)	5	78.6 (0.686–0.886)	760	91.19	<0.001	0.0013
Study year (2001–2020)	14	49.7 (0.399–0.595)	8854	98.79	<0.001	0.8133

In studies with both children and adolescents and adults as respondents, data were extracted by segregating the results according to two age groups, if possible.

CI, confidence interval.

#### *Publication bias*

**Figure 5:**
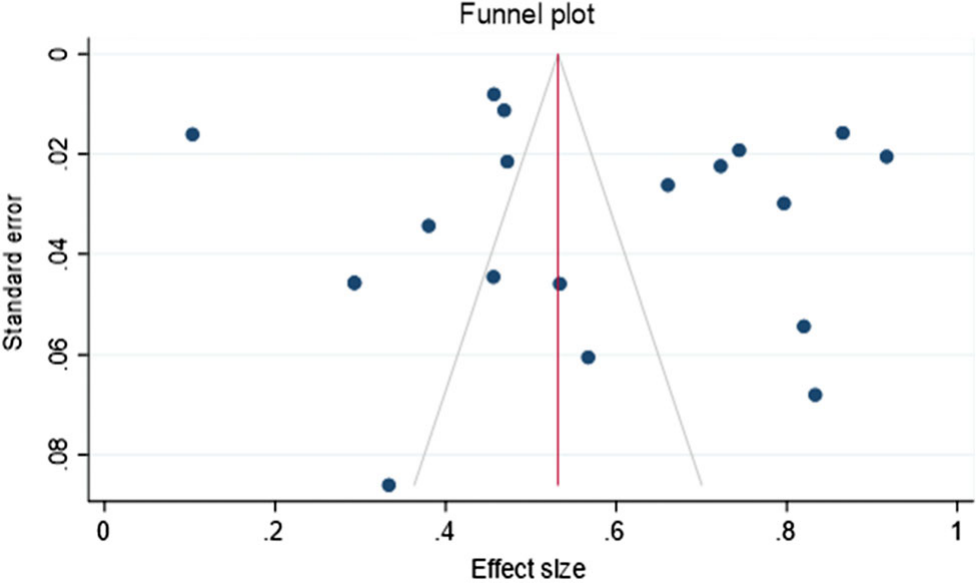
Symmetrical funnel plot of standard error with effect size visualizing 19 studies. (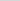), Pseudo 95% confidence interval; (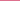), estimated θ_IV_; (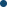), studies.

Funnel plots of standard error with effect size and linear regression test for small study effect size confirmed no publication bias in all the 19 included studies reporting prevalence of *H. pylori* infection (Eggers' test: *P* = 0.747) as shown in Figure 5.

No publication bias was observed among 11 studies showing the prevalence of *H. pylori* between males (*P* = 0.4944) and females (*P* = 0.6898). Similar results were obtained in 9 studies showing prevalence among children and adolescents (*P* = 0.4038), 7 studies among adults (*P* = 0.5312), and 14 studies from 2000 to 2020. (*P* = 0.8133).

## Discussion

The present study is the first systematic review and meta‐analysis on the prevalence of *H. pylori* infection among asymptomatic individuals in South Asia. This has shown a wide variation in *H. pylori* infection among asymptomatic healthy populations across different South Asian countries.

A study by Hooi *et al*. about worldwide *H. pylori* prevalence showed a worldwide pooled prevalence of 63.4%, 61.6% in the southern Asian region, 43.1% in the southeastern Asian region, 66.6% in the western Asian region, 54.1% in the eastern Asian region, and 79.5% in the central Asian region.^2^ Another review by Zamani *et al*. showed a worldwide prevalence of 44.3 and 55% to more than 70% in South Asian countries.^14^

In our systematic review and meta‐analysis, the pooled prevalence obtained was 56.5%, ranging from 10.3 to 91.7%. In comparison to different regions of Asia, the prevalence in our study in South Asian countries is higher than in southeastern Asian and eastern Asian regions and lower than the central and western regions of Asia. A study in central Asia concluded poor sanitation and low hygiene practices, low economic condition, and impure water as likely reasons for higher *H. pylori* prevalence in that region.^20^ In addition to the aforementioned risk factors prevalent in South Asian countries, poor parental education and unhealthy habits like smoking, drinking alcohol, and eating raw vegetables and spicy food contribute to high *H. pylori* infection, practices similar to Iran and the East Mediterranean region, which also have high *H. pylori* prevalence among healthy populations.^21^ In contrast, industrialized and developed countries in eastern Asia with higher socioeconomic and education status and a healthy lifestyle and countries in southeastern Asia have lower *H. pylori* prevalence.^22^, ^23^

Country‐wise pooled prevalence of *H. pylori* infection in our subgroup analysis showed the lowest prevalence of 10.3% in Sri Lanka and the highest prevalence of 86.3% in Bangladesh, with Pakistan at 56.6% and India at 49.5%. The low prevalence in Sri Lanka could be due to the use of conventional *H. pylori* diagnostic methods to obtain negative serum samples in addition to the high cut‐off point.^21^The differences in prevalence among different countries may be due to strain differences or various virulence factors of *H. pylori*, genetic differences of the host, environmental conditions, and household hygiene practices among different populations of the countries of South Asia.

Gender‐wise pooled prevalence of *H. pylori* infection in our subgroup analysis showed no differences between the two, suggesting similar behavioral factors among males and females, which is similar to the previous review by Zamani *et al*.^14^ Systematic review and meta‐analysis of 244 studies by Ibrahim *et al*. showed a male predominance in *H. pylori* prevalence in both pediatric and adult populations, but the association was stronger in adults than in children.^24^ However, the relationship between *H. pylori* infection and gender remains a topic of discussion.^25^ A male predominance is seen in gastric adenocarcinoma, and gender as a biological risk factor should be considered for *H. pylori* infections and complications while screening the population.^14^

The age‐wise pooled prevalence of *H. pylori* infection showed a higher prevalence in children and adolescents than adults. A substantial increase in infection rate from a baseline of 0.6% in the newborn to 13.5% in high school students and a maximum reaching up to 33% was reported in China.^26^ Differences in the infection time, transmission route, pathogenicity, and drug susceptibility among children, adolescents, and adults may be considered the main reasons.^27^ Moreover, the majority of *H. pylori* infections are mainly acquired in childhood.^12^ A birth cohort study is needed in this region to explore associations between drug susceptibility, transmission route, infection time, and *H. pylori* infection. This study shows a decreasing trend of *H. pylori* infection in South Asia in the past 30 years: 78.6% (before 2000) and 49.7% (2001–2020). Similar results are shown by different studies conducted in Asia.^14^, ^24^ Increased health‐seeking attitude and use of triple‐drug therapy for eradication of *H. pylori* along with improvements in socioeconomic status and lifestyles of people may be the contributing factors of a fall in *H. pylori* infection rates over the years.

In the diagnosis of *H. pylori*, sensitivity and specificity of different methods and assays may be considered limiting factors for comparison, so subgroup analysis was not done.^2^ Similarly, accessibility and cost‐effectiveness of different methodologies in different parts of the world, such as serology, that are used in mainly Asian studies may influence the outcome.^14^ *H. pylori* serology is used in many studies in our analysis but is not a reliable method as it detects antibody for a long time even after successful eradication.^28^

*H. pylori* is currently the most important controllable factor for preventing gastric cancer.^29^ Several studies have provided evidence that treating and eradicating *H. pylori* infection among asymptomatic individuals reduces the risk of gastric cancer and is a cost‐effective option.^30^, ^31^, ^32^ This emphasizes the importance of timely diagnosing and treating asymptomatic *H. pylori* infection for reducing gastric cancer risk. Meanwhile, eradication therapy for positive but asymptomatic individuals is more economical and more effective than no eradication treatment.^29^ Thus, it seems likely that the benefit of searching for and eradicating *H. pylori* in healthy asymptomatic individuals will outweigh any potential harms, especially in populations at high risk of gastric cancer.^31^

Although, the “test‐and‐treat” approach for *H. pylori* is introduced, which reduces underlying peptic ulcer disease and gastroduodenal diseases,^33^ its beneficial effect on gastric cancer reduction is not elucidated. In addition, mass screening for *H. pylori* infection and eradication of the infection in positive healthy individuals is still dubious due to the increased risk of antibiotic resistance, eradication failure, and risk of developing esophageal adenocarcinoma in the long term.^34^, ^35^, ^36^, ^37^, ^38^

Our study provides a platform for further studies on healthy/asymptomatic populations on *H. pylori* prevalence in this region. A large multicentric cohort study is necessary in this part of the world to know the benefit of timely screening and treatment of *H. pylori* infection among asymptomatic populations to reduce gastric cancer cases, prevent other gastroduodenal diseases, and demonstrate the usefulness of *H. pylori* eradication therapy.

There are some limitations to our study. The age range of participation was wider, which caused difficulty in comparison of studies. Significant heterogeneity was found in general and within each subgroup, a; though South Asians are almost identical in race, economy, health‐care status, education, and culture. Variation in the design of observational studies and baseline characteristics of included healthy individuals, such as participants of different age group, ethnicity, lifestyles, and use of different diagnostic tests with different sensitivity and specificity, were possible factors for high heterogeneity. Due to irretrievable full texts and insufficient data in some studies of *H. pylori* prevalence, the effect of these studies on pooled prevalence was not calculated. Confounders of *H. pylori* infection, such as smoking, drinking, and dietary factors, were not considered in our study.

## Conclusions

This meta‐analysis identified a high prevalence of *H. pylori* infection among asymptomatic healthy populations of South Asia, particularly in children and adolescents. However, with improved socioeconomic conditions and hygiene, decreasing rates of *H. pylori* infection are reported from many regions worldwide. Thus, improving water quality, sanitation, personal hygiene, and access to health‐care facilities along with timely screening may be a beneficial and cost‐effective measure in decreasing the burden of *H. pylori* infection and its consequences in the developing nations of South Asia. The high burden of asymptomatic *H. pylori* infection among both children and adults warrants the need for more reviews and studies focusing on the effect of asymptomatic infection on the global burden of upper gastrointestinal diseases, the evolution of dyspeptic symptoms and strategies for its prevention, and the possible need of vaccination.

1

## Supporting information


**Appendix** **S1.** Supplementary Information.

## Data Availability

The data that support the findings of this study are available from the corresponding author upon reasonable request.
