# Supplementary material for: Helicobacter pylori healthy South Asians
Source: JGH Open. 2020 Oct 12;4(6):1037–46. doi: 10.1002/jgh3.12426 (PMC7731813; doi:10.1002/jgh3.12426)
Supplement: Supplementary file 1 — Appendix S1. Supplementary Information. [file JGH3-4-1037-s001.docx]

Supplementary Content

# Prevalence of Helicobacter Pylori Infection Among the Healthy Population in South Asia:

**A Systematic Review and Meta-analysis**

**Appendix 1:** Search strategy used in the current systematic review and meta- analysis.

**Appendix 2:** Newcastle-Ottawa Scale for cross-sectional studies and case-control studies

**Appendix 3:** Quality assessment of the included articles

**Appendix 4:** Sensitivity Analysis by omitting one study at a time using random effect model

This supplementary material has been provided by the authors to give readers additional information.

**Appendix 1:** Search strategy used in the current systematic review and meta- analysis.

For PubMed

#1: "Helicobacter pylori"[Mesh Terms]

#2: "Helicobacter infection"[Mesh Terms]

#3: #1 OR #2

#4: ("Prevalence"[Mesh] OR "Epidemiology"[Mesh] OR "Seroprevalence")

#5: #3 AND #4

#6: (“Afghanistan” OR “Bangladesh” OR “Bhutan” OR “India” OR “Maldives” OR “Nepal” OR “Pakistan” OR “Srilanka”)

#7: #5 AND #6

Filters: human subjects, English language, Time frame from 1983/06/01 to 2020/05/01

# Final search strategy after filters

(((((("Helicobacter pylori"[Mesh]) OR "Helicobacter Infections"[Mesh]) AND (("Prevalence"[Mesh]) OR "Epidemiology"[Mesh] OR "Seroprevalence")) AND (“Afghanistan” OR “Bangladesh” OR “ Bhutan” OR “ India” OR “ Maldives” OR “Nepal” OR “ Pakistan” OR “Srilanka”) ] AND ("1983/06/1"[PDAT] : "2020/05/1"[PDAT]) AND "humans"[Mesh Terms] AND English[Lang])

**Appendix 2:** Newcastle-Ottawa Scale for cross-sectional studies and case-control

studies

# NEWCASTLE - OTTAWA QUALITY ASSESSMENT SCALE

**(adapted for cross sectional studies)**

**Selection:** (Maximum 5 stars)

1. Representativeness of the sample:
2. Truly representative of the average in the target population. * (all subjects or random sampling)
3. Somewhat representative of the average in the target population. * (non-random sampling)
4. Selected group of users.
5. No description of the sampling strategy.
6. Sample size:
   1. Justified and satisfactory. *
   2. Not justified.
7. Non-respondents:
8. Comparability between respondents and non-respondents’ characteristics is established, and the response rate is satisfactory. *
9. The response rate is unsatisfactory, or the comparability between respondents and non-respondents is unsatisfactory.
10. No description of the response rate or the characteristics of the responders and the non-responders.
11. Ascertainment of the exposure (risk factor):
    1. Validated measurement tool. **
    2. Non-validated measurement tool, but the tool is available or described. *
    3. No description of the measurement tool.

**Comparability:**(Maximum 2 stars)

1) The subjects in different outcome groups are comparable, based on the study design or analysis. Confounding factors are controlled.

1. The study controls for the most important factor (select one). *
2. The study control for any additional factor. *

**Outcome:** (Maximum 3 stars)

- 1. Assessment of the outcome:
     1. Independent blind assessment. **
     2. Record linkage. **
     3. Self-report. *
     4. No description.
  2. Statistical test:

1. The statistical test used to analyze the data is clearly described and appropriate, and the measurement of the association is presented, including confidence intervals and the probability level (p value). *
2. The statistical test is not appropriate, not described or incomplete.

**NEWCASTLE - OTTAWA QUALITY ASSESSMENT SCALE**

**(**Case Control Studies)

**Selection**

- 1. Is the case definition adequate?
     1. yes, with independent validation *****
     2. yes, e.g. record linkage or based on self-reports
     3. no description
  2. Representativeness of the cases
     1. consecutive or obviously representative series of cases *****
     2. potential for selection biases or not stated
  3. Selection of Controls
     1. community controls *****
     2. hospital controls
     3. no description
  4. Definition of Controls
     1. no history of disease (endpoint) *****
     2. no description of source

# Comparability

- - - 1. Comparability of cases and controls on the basis of the design or analysis
         1. study controls for (Select the most important factor.) *****
         2. study controls for any additional factor ***** (These criteria could be modified to indicate specific control for a second important factor.)

**Exposure**

1. Ascertainment of exposure
   1. secure record (eg surgical records) *****
   2. structured interview where blind to case/control status *****
   3. interview not blinded to case/control status
   4. written self-report or medical record only
   5. no description
2. Same method of ascertainment for cases and controls
   1. yes *****
   2. no
3. Non-Response rate
   1. same rate for both groups *****
   2. non respondents described
   3. rate different and no designation

Note: A study can be awarded a maximum of one star for each numbered item within the Selection and Exposure categories. A maximum of two stars can be given for Comparability.

In this scale, the scoring ranges from 0 to 10. Studies with mean scores greater than or equal to seven were considered as “low risk” while studies with a mean score less than seven were considered “high risk”. All the studies with mean scores greater than equal to 5 are included in the analysis.

**Appendix 3:** Quality assessment of the included article

| Study Name | Author (SK) | Author (AB) | Mean Score | Risk of Bias | Included/ Excluded |
| --- | --- | --- | --- | --- | --- |
| Ahmad 1997^1^ | 6 | 6 | 6 | High | Included |
| Ahmad 2008^2^ | 8 | 7 | 7.5 | Low | Included |
| Augustine 2019^3^ | 5 | 5 | 5 | High | Included |
| Dore 1997^4^ | 5 | 5 | 5 | High | Included |
| Fernando 2003^5^ | 7 | 7 | 7 | Low | Included |
| Jafri 2009^6^ | 8 | 8 | 8 | Low | Included |
| Jafri 2013^7^ | 8 | 8 | 8 | Low | Included |
| Mahskar 2010^8^ | 6 | 6 | 6 | High | Included |
| Mehmood 2014^9^ | 5 | 5 | 5 | High | Included |
| Prasad 1994^10^ | 6 | 6 | 6 | High | Included |
| Priyadarshani 2018^11^ | 6 | 6 | 6 | High | Included |
| Rasheed 2011^12^ | 7 | 7 | 7 | Low | Included |
| Rifat-uz-Zaman 2006^13^ | 7 | 7 | 7 | Low | Included |
| Romshoo 1997^14^ | 5 | 5 | 5 | High | Included |
| Sarker 1997^15^ | 7 | 8 | 7.5 | Low | Included |
| Sarker 2004^16^ | 6 | 6 | 6 | High | Included |
| Singh 2002^17^ | 8 | 8 | 8 | Low | Included |
| Tewari 2012^18^ | 7 | 7 | 7 | Low | Included |
| Wangda 2017^19^ | 8 | 8 | 8 | Low | Included |
|  |  |  | Kappa value=0.859259 Strong  Agreement |  |  |

# Note: Mean scores greater or equal to 5 are included in analysis.

**Appendix 4:** Sensitivity Analysis by omitting one study at a time using random effect model

| Author Year | Prevalence  (%) | Lower  95% C.I. | Upper  95% C.I. | Tau ^2^ | I2 |
| --- | --- | --- | --- | --- | --- |
| Omitting Ahmad 1997^1^ | 54.5 | 0.444 | 0.646 | 0.0460 | 99.00 |
| Omitting Ahmad 2008^2^ | 55.6 | 0.447 | 0.664 | 0.0535 | 99.15 |
| Omitting Augustine 2019^3^ | 58.0 | 0.472 | 0.687 | 0.0524 | 99.17 |
| Omitting Dore 1997^4^ | 55.1 | 0.444 | 0.658 | 0.0522 | 99.17 |
| Omitting Fernando 2003^5^ | 59.2 | 0.501 | 0.682 | 0.0365 | 98.69 |
| Omitting Jafri 2009^6^ | 57.0 | 0.451 | 0.689 | 0.0644 | 99.16 |
| Omitting Jafri 2013^7^ | 57.0 | 0.459 | 0.681 | 0.0556 | 99.18 |
| Omitting Mahskar 2010^8^ | 57.1 | 0.463 | 0.679 | 0.0531 | 99.18 |
| Omitting Mehmood 2014^9^ | 56.7 | 0.458 | 0.675 | 0.0531 | 99.18 |
| Omitting Prasad 1994^10^ | 55.1 | 0.444 | 0.658 | 0.0522 | 99.17 |
| Omitting Priyadarshani 2018^11^ | 58.0 | 0.472 | 0.687 | 0.0524 | 99.17 |
| Omitting Rasheed 2011^12^ | 55.5 | 0.446 | 0.663 | 0.0532 | 99.12 |
| Omitting Rifat-uz-Zaman 2006^13^ | 57.1 | 0.447 | 0.694 | 0.0698 | 99.12 |
| Omitting Romshoo 1997^14^ | 57.6 | 0.469 | 0.684 | 0.0525 | 99.18 |
| Omitting Sarker 1997^15^ | 54.8 | 0.448 | 0.647 | 0.0449 | 98.92 |
| Omitting Sarker 2004^16^ | 55.2 | 0.449 | 0.659 | 0.0520 | 99.15 |
| Omitting Singh 2002^17^ | 56.5 | 0.457 | 0.672 | 0.0528 | 99.18 |
| Omitting Tewari 2012^18^ | 57.5 | 0.467 | 0.683 | 0.0532 | 99.17 |
| Omitting Wangda 2017^19^ | 55.9 | 0.450 | 0.668 | 0.0540 | 99.17 |
| Total | 56.5 | 0.460 | 0.669 | 0.0524 | 99.13 |

**REFRENCES** H

1. Ahmad MM, Rahman M, Rumi AK, et al. Prevalence of Helicobacter pylori in asymptomatic population--a pilot serological study in Bangladesh. J Epidemiol. 1997;7(4):251-254. doi:10.2188/jea.7.251
2. Ahmad T, R Bilal, A Khanum. Prevalence of Helicobacter pylori infection in asymptomatic children of Islamabad suburbs (Pakistan). Int J Agri Biol. 2008;10:685–8. [[Full Text](http://www.fspublishers.org/published_papers/81252_..pdf)]
3. P Augustine J, Microbiology. Prevalence of asymptomatic Helicobacter pylori infection in Kerala, India. IJMR. 2019;6(2):180-183. doi:10.18231/j.ijmr.2019.039
4. Fernando N, Weerasekera D, Fernando S, Liyanage N, Holton J. Helicobacter pylori serology in two MOH areas of the Western Province of Sri Lanka. Cey J Med Sci. 2003;46(2):35. doi:10.4038/cjms.v46i2.4827
5. Dore SP, Krupadas S, Borgonha S, Kurpad AV. The 13C urea breath test to assess Helicobacter pylori infection in school children. The National Medical Journal of India. 1997;10(2):57-60 [[Full Text](https://europepmc.org/article/med/9153979)]

1. Jafri W, Yakoob J, Abid S, Siddiqui S, Awan S, Nizami SQ. Helicobacter pylori infection in children: population-based age-specific prevalence and risk factors in a developing country. Acta Paediatr. 2010;99(2):279-282. doi:10.1111/j.1651-2227.2009.01542.x
2. Jafri W, Yakoob J, Abid S, et al. Seroprevalence of hepatitis E and Helicobacter pylori in a low socioeconomic area of a metropolitan city in a developing country. Br J Biomed Sci. 2013;70(1):27-30. doi:10.1080/09674845.2013.11669926
3. Mhaskar RS. Epidemiological Study of Contributing Factors in the Development of Peptic Ulcer and Gastric Cancer Initiated by Helicobacter Pylori Infection in India. 2010. https://scholarcommons.usf.edu/etd/3493/. Accessed July 28, 2020.
4. Mehmood K. Lower Education Status Predicts Higher Seropositivity for Helicobacter pylori Infection in Pakistan. ARRB. 2014;4(24):3734-3741. doi:10.9734/ARRB/2014/10788
5. Prasad S, Mathan M, Chandy G, et al. Prevalence of Helicobacter pylori in southern Indian controls and patients with gastroduodenal disease. J Gastroenterol Hepatol. 1994;9(5):501-506. doi:10.1111/j.1440-1746.1994.tb01281.x
6. Priyadarshini DR, M. Easow J, Vinod R. Seroprevalence of Helicobacter pylori among Healthy Blood Donors. IntJCurrMicrobiolAppSci. 2018;7(2):817-822. doi:10.20546/ijcmas.2018.702.103
7. Rasheed F, Ahmad T, Bilal R. Frequency of Helicobacter pylori infection using 13C-UBT in asymptomatic individuals of Barakaho, Islamabad, Pakistan. J Coll Physicians Surg Pak. 2011;21(6):379-381. doi:07.2011/JCPSP.379381
8. R-U-Z. Prevalence of Helicobacter pylori in Relation to Promotive Factors among Human Urban Population of Bahawalpur District, Pakistan. Pakistan Journal of Biological Sciences. 2006;9(14):2636-2641. doi:10.3923/pjbs.2006.2636.2641
9. Romshoo GJ, Bhat MY, Malik GM, et al. Helicobacter pylori Infection in Various ABO Blood Groups of Kashmiri Population. Diagn Ther Endosc. 1997;4(2):65-67. doi:10.1155/DTE.4.65
10. Sarker SA, Mahalanabis D, Hildebrand P, et al. Helicobacter pylori: prevalence, transmission, and serum pepsinogen II concentrations in children of a poor periurban community in Bangladesh. Clin Infect Dis. 1997;25(5):990-995. doi:10.1086/516070
11. Sarker SA, Nahar S, Rahman M, et al. High prevalence of cagA and vacA seropositivity in asymptomatic Bangladeshi children with Helicobacter pylori infection. Acta Paediatr. 2004;93(11):1432-1436. doi:10.1080/08035250410033088
12. Singh V, Trikha B, Nain CK, Singh K, Vaiphei K. Epidemiology of Helicobacter pylori and peptic ulcer in India. J Gastroenterol Hepatol. 2002;17(6):659-665. doi:10.1046/j.1440-1746.2002.02746.x
13. Tewari R, Nijhawan VS, Mishra MN, Dudeja P, Salopal TK. Prevalence of Helicobacter pylori, cytomegalovirus, and Chlamydia pneumoniae immunoglobulin seropositivity in coronary artery disease patients and normal individuals in North Indian population. Armed Forces Med J India. 2012;68(1):53-57. doi:10.1016/S0377-1237(11)60121-4
14. Wangda S, Richter JM, Kuenzang P, et al. Epidemiology of Helicobacter pylori infection in asymptomatic schoolchildren in Bhutan. Helicobacter. 2017;22(6). doi:10.1111/hel.12439
